# Neonatal resuscitation workshop for trainees in standardized medical residency training—a pilot practice in Shenzhen, China

**DOI:** 10.3389/fped.2023.1237747

**Published:** 2023-09-04

**Authors:** Chenguang Xu, Qianshen Zhang, Yin Xue, Yuqian Yang, Yihua Chen, Wenjie Yan, Po-Yin Cheung

**Affiliations:** ^1^ NICU, The University of Hong Kong-Shenzhen Hospital, Shenzhen, China; ^2^ Centre for the Studies of Asphyxia and Resuscitation, Neonatal Research Unit, Royal Alexandra Hospital, Edmonton, AB, Canada; ^3^ NICU, University of Alberta, Edmonton, AB, Canada

**Keywords:** neonatal resuscitation, simulation, workshop, residency training, healthcare professional (HCP)

## Abstract

**Background:**

Neonatal resuscitation is an important skillset for clinicians attending deliveries. Accredited neonatal resuscitation training is not obligatory in most training centers of standardized medical residency programs before 2022 in China. We investigated the feasibility and effectiveness of neonatal resuscitation simulation training (neo-RST) in residents in Shenzhen, China.

**Methods:**

Four two-day neo-RST workshops were conducted in the University of Hong Kong-Shenzhen Hospital and Shenzhen Health Capacity Building and Continuing Education Center in 2020–2021. The workshops had Neonatal Resuscitation Program (NRP)® update, skill stations and simulation practice with debriefing. Each participant had the integrated skill station assessment (ISSA) at the end of workshop. Participants of workshops included residents of different disciplines and health care providers (HCPs) of neonatal and obstetrical departments. We compared demographic characteristics, neonatal resuscitation knowledge before training, ISSA overall and categorical scores on skill sets between residents and HCPs.

**Results:**

In 2020–2021, 4 neo-RST workshops were conducted with 48 residents and 48 HCPs. The residents group had less working experience, less prior experience in neo-RST and lower neonatal resuscitation knowledge scores than those of HCPs group. After the workshop, residents had higher overall ISSA score than that of HCPs group (90.2 ± 5.9 vs. 86.3 ± 6.6%, *P *= 0.003, respectively). There was no significant difference in the numbers of participants scored <80% in residents and HCPs group (3 [6.3%] vs. 7 [14.6%], respectively). Regarding the categorical scores, residents scored significantly higher in preparation, ventilation, crisis resource management and behavioral skills but lower in appropriate oxygen use, when compared with the HCPs.

**Conclusion:**

Neo-RST for residents is feasible with promising short-term educational outcomes. Neo-RST could be implemented in standardized medical residency programs in China.

## Introduction

Most neonates can successfully transition to extrauterine life without assistance, but about 10% of newborns need some degree of resuscitation to establish breathing (1). The large number of births every year means that skilled providers, who are competent in neonatal resuscitation, must be ready to make a quick and effective response to each newborn (2). Therefore, neonatal resuscitation is an important skillset for all clinicians attending deliveries.

Neonatal Resuscitation Program® (NRP®) was developed by the American Academy of Pediatrics (AAP) and the American Heart Association (AHA) based on the latest scientific evidence, with the goal of having at least one trained provider present at each delivery (3). In North America, pediatric residents are required to complete training in NRP® according to the Accreditation Council for Graduate Medical Education (ACGME) (4, 5). In most developed countries and some developing countries, NRP® provider training or its equivalent is mandatory for pediatric residents and/or medical interns during their rotation through neonatal units (6, 7). However, standardized training in neonatal resuscitation is not mandatory in most training centers in China before 2022.

There has been remarkable improvement in medical education in China for the past 10 years (8, 9). Resident training has been developed rapidly since the introduction of “Standardized Residency Training in China” in December 2013 (10). While NRP® is not obligatory in the national training guidelines for standardized medical residency programs in China in 2015 version (11), the Chinese Medical Association revised the training guidelines with detailed competency requirement in August 2022 (12). The guidelines set the requirement for comprehensive competency of residents and proposed the requirement of neonatal resuscitation skills for pediatric residents in this version. Effectiveness of simulation-based neonatal resuscitation among pediatric residents or medical students in China has been reported in areas with advanced medical resources and excellent education in China, such as Beijing and Shanghai (13, 14). It is uncertain if simulation-based neonatal resuscitation training would develop the competency of residents in neonatal resuscitation skills in other areas, especially in regions where educational resources are limited or medical heritage is lacking.

We aimed to investigate the feasibility and effectiveness of the neonatal resuscitation simulation training (neo-RST) in residents in Shenzhen, China. We hypothesized that after standardized neo-RST workshops, residents could achieve the competence in simulated neonatal resuscitation assessment, comparable to that of health care providers (HCPs) in neonatal intensive care unit (NICU) and obstetric unit.

## Methods

The University of Hong Kong-Shenzhen Hospital (HKU-SZH) is a comprehensive hospital, which has adopted evidence-based practices and neo-RST workshops to provide training to NICU and obstetric staff in HKU-SZH since 2013 and to HCPs nationwide since 2016. Since 2018, HKU-SZH has become one of the standardized residency training centers in China.

Four neo-RST workshops for providers were conducted in HKU-SZH and Shenzhen Health Capacity Building and Continuing Education Center in 2020–2021. The curriculum was based on the 2015 NRP® algorithm, 7th edition of NRP® textbook with modifications for the context in China (1, 15, 16). There were 24 participants taught by 6 instructors (all from NICU, HKU-SZH) in each workshop with the ratio of instructors to providers was 1:4.

**Figure 1:**
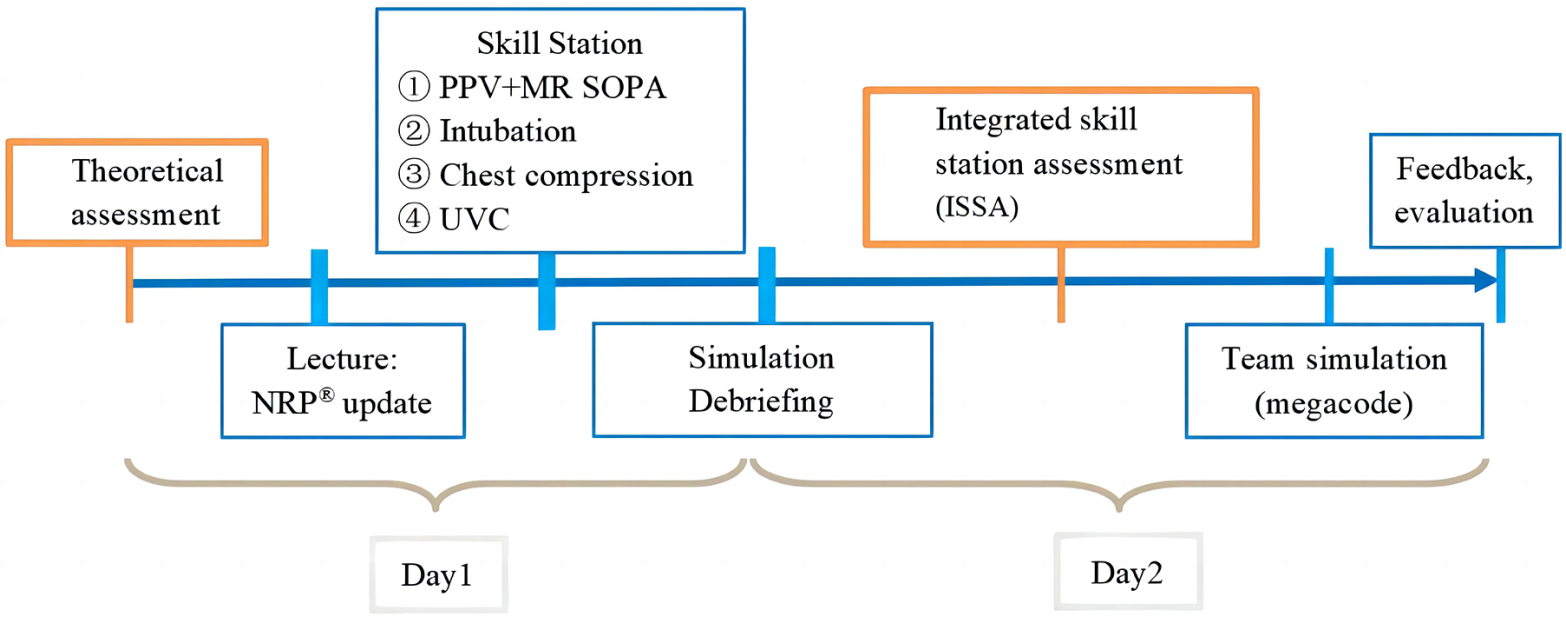
Training process of neonatal resuscitation workshop.

Before the workshop, each participant was required to study 7th edition of NRP® textbook independently (15), completed and passed an examination composed of multiple-choice questions in neonatal resuscitation with a score of ≥80%. The 2-day workshops started with a didactic lecture, followed by four skill stations including positive pressure ventilation (PPV) and ventilation corrective steps (MR SOPA: Mask adjustment, Reposition of the airway, Suction the mouth and nose, Opening mouth, increasing Pressure and placing Alternative airway), intubation, chest compression (CC) and umbilical vein catheterization (UVC). Each skill station included cognitive, technical and behavioral objectives, with technical skills being the most important goal. The technical skills were demonstrated by instructors first, then the participants practiced the skills with coaching by instructors. After that, repeated simulations with debriefing were practiced to integrate these skills into resuscitation. Laerdal® SimNewB^™^ high fidelity manikin was used together with simulated clinical scenario in simulation center (Figure 1 and Table 1). Most but not all the clinical scenarios were related to the neonatal resuscitation at delivery.

**Table 1:** Objectives of four skill stations in neo-RST workshops.

Objectives	PPV + MR SOPA	Intubation	Chest compression (CC)	UVC
Cognitive	☑ Know airway management in neonatal resuscitation ☑ Identify indications for PPV	☑ Identify indications for intubation	☑ Identify indications for starting and stopping CC ☑ Know when to reassess heart rate after commencing CC	☑ Identify indications for medication and UVC placement ☑ Know the route and doses of medication
Technical	☑ Correctly perform PPV and corrective ventilation steps MR SOPA	☑ Correctly perform endotracheal intubation ☑ Determine the position of endotracheal tube	☑ CC with correct position, depth, rebound, frequency, FiO2, time ☑ Cooperation between CC and ventilation	☑ Prepare for emergency UVC and medication ☑ Identify umbilical vessels and place catheter correctly
Behavioral	☑ Effective teamwork and communication ☑ Use available resources	☑ Effective teamwork and communication	☑ Effective teamwork and communication	☑ Know environment ☑ Use available information ☑ Effective teamwork and communication

Each participant would have the integrated skill station assessment (ISSA) after repeated simulation practice. The ISSA form was based on a performance checklist developed by Lockyer et al. to assess neonatal resuscitation megacode skill (17). The ISSA form adopted by the Canadian NRP® (Canadian Paediatric Society, November 15, 2016 version) was used and translated into Chinese (Supplementary Materials). During the assessment, two participants worked as a team and alternated as the leader and assistant to complete a resuscitation scenario for each participant. Each scenario lasted for 8–10 min and the performance of participants was assessed by two instructors. The participants were expected to ask questions to identify risk factors, predict possible actions, propose an appropriate plan, give clear role assignment and check equipment before delivery. After the simulated baby was born, the participants would be evaluated in real time and perform appropriate resuscitation steps in the context of the scenarios following 2015 NRP® algorithm (1). The overall neonatal resuscitation competence was assessed with a primary focus on technical skills and its integration in the resuscitation procedures and a secondary focus on behavioral skills. Two standardized scenarios were used alternatively in each workshop for the assessment of integrated skills in the resuscitation of full-term or late preterm neonate with fetal distress at birth. There were two types of ISSA including advanced and basic types. The categorical items in advanced ISSA included (1) preparation (know the environment, use available information, anticipate and plan, clearly identify a team leader; 6 points), (2) initial steps (8 points), (3) ventilation (14 points): PPV and MR SOPA (12 points) and intubation (2 points), (4) chest compression (6 points), (5) medication (4 points), (6) appropriate oxygen use (6 points), (7) crisis resources management (CRM, 2–4 points). The assessment of CRM included the ability to call for help if needed, to identify additional interventions as indicated on the history and clinical response to resuscitation. Intubation and medication were not included in basic ISSA. The full original score for advanced providers was 46–48 in different scenarios with 2 additional points for special considerations for preterm neonates. As NICU nurses and midwives were not required to perform intubation in China, the respective full original score for them as basic providers was 40–42, whereas 46–48 for physicians. The total original score was also converted to percentages with a passing score of 80%. If the participant did not meet the expected requirements, s/he would receive short-term coaching and remedial training, followed by a supplementary evaluation by another instructor.

Team resuscitation training (megacode) was performed after ISSA with the primary goal of teamwork and behavioral skills training (15). Audio and video assistance was provided during team simulation and debriefing with informed consents from all participants.

Participants of workshops included residents (less than three years clinical experience) and HCPs of NICU and obstetrical unit. Residents were recruited from 6 training centers in Shenzhen, with the majority coming from pediatric departments. The neo-RST workshops designed for residents were first of this kind and non-mandatory with voluntary enrollment.

For this educational study, informed consents were obtained from all participants prior to the start of neo-RST workshops. The demographic characteristics of participants including working and simulation training experience, neonatal resuscitation knowledge before training were obtained and compared between residents and HCPs groups. The educational outcomes after workshops included overall ISSA and categorical scores in the first assessment. We further analyzed behavioral skills scores by combining categorical scores in preparation and CRM. All data were prospectively collected in training records.

### Statistical analyses

Data was presented in mean ± SD or median and IQR for parametric and non-parametric variables, respectively. Differences between two groups were analyzed by Student's *t*-test for continuous parametric variables and Chi-square test or Fisher Exact test as appropriate for categorical variables. SPSS Statistics (v.23) was used for data analyses. A *P*-value less than 0.05 was considered statistically significant.

## Results

Of 4 neo-RST workshops conducted in 2020–2021, two were specially conducted for residents. There were 48 residents from six training centers with standardized medical residency programs in Shenzhen in the residents group and 48 HCPs from HKU-SZH and other centers in China. In the residents group, 34 were pediatric residents and 14 were from obstetrics and gynecology (7), anesthesiology (4) and emergency (3) departments. There were 31 physicians, 13 NICU nurses and 4 midwives in the HCPs group.

The residents group had less working experience (1.9 ± 0.5 years vs. 6.6 ± 5.2 years of HCPs group, *P *< 0.001). Only 19% of residents had prior experience in neo-RST, whereas 44% of HCPs had ever participated in neo-RST (*P *= 0.008). Prior to neo-RST workshops, residents had lower neonatal resuscitation knowledge scores than those of HCPs group (76.1 ± 13.6 vs. 89.5 ± 9.0, *P *< 0.001, respectively), only 23 (47.9%) residents got MCQ scores above 80% [vs. 42 (87.5%) HCPs, *P *< 0.001]. There was no difference in the proportion of gender, pediatricians and whether delivery room was available in respective hospitals (Table 2).

**Table 2:** Demographic characteristics and neonatal resuscitation performance as indicated by ISSA overall and categorical scores of 48 residents and 48 health care professionals (HCPs) groups.

		Residents group	HCPs group	*P-*value
Demographic characteristics				
Work experience (year)		1.9 ± 0.5	6.6 ± 5.2	<0.001
Delivery room available		41 (85%)	44 (92%)	0.336
Female gender		37 (77%)	33 (69%)	0.358
Proportion of pediatric physicians		34 (71%)	31 (65%)	0.513
Previous participation in neonatal resuscitation simulation training		9 (19%)	21 (44%)	0.008
Neonatal resuscitation knowledge prior to workshop (% score)		76.1 ± 13.6	89.5 ± 9.0	<0.001
Neonatal resuscitation performance				
Overall ISSA score (first assessment)	[%]	90.2 ± 5.9%	86.3 ± 6.6%	0.003
	*n* (%) with score <80%	3 (6.3%)	7 (14.6%)	0.181
Preparation	Original score (full score* *= 6) [%]	5.8 ± 0.5 [96.2 ± 8.6%]	4.9 ± 0.8 [81.6 ± 13.4%]	<0.001
	*n* (%) with score <80%	2 (4.2%)	14 (29.2%)	0.001
Initial steps	Original score (full score* *= 8) [%]	7.3 ± 0.8 [91.1 ± 10.6%]	7.0 ± 0.9 [88.0 ± 11.2%]	0.164
	*n* (%) with score <80%	8 (16.7%)	11 (22.9%)	0.442
Ventilation (basic): PPV + MR SOPA	Original score (full score* *= 12) [%]	10.9 ± 0.9 [91.0 ± 7.3%]	10.6 ± 1.4 [88.0 ± 11.7%]	0.140
	*n* (%) with score <80%	3 (6.3%)	10 (20.8%)	0.037
Intubation^a^	Original score (full score* *= 2) [%]	1.8 ± 0.4 [91.7 ± 18.8%]	1.7 ± 0.5 [87.1 ± 25.3%]	0.353
	*n* (%) with score <80%	8 (16.7%)	8 (22.9%)	0.480
Ventilation (advanced)^a^: PPV + MR SOPA + intubation	Original score (full score* *= 14) [%]	12.8 ± 0.9 [91.1 ± 6.5%]	12.1 ± 1.6 [86.5 ± 11.3%]	0.024
	*n* (%) with score <80%	5 (10.4%)	11 (31.4%)	0.017
Chest compression	Original score (full score* *= 6) [%]	5.2 ± 1.2 [86.1 ± 20.1%]	5.3 ± 0.8 [87.8 ± 13.2%]	0.619
	*n* (%) with score <80%	8 (16.7%)	8 (16.7%)	1.000
Medication^a^	Original score (full score* *= 4) [%]	3.8 ± 0.5 [94.3 ± 12.9%]	3.8 ± 0.6 [94.3 ± 15.0%]	0.996
	*n* (%) with score <80%	9 (18.8%)	5 (14.3%)	0.592
Appropriate oxygen use	Original score (full score* *= 6) [%]	4.9 ± 1.0 [81.3 ± 17.0%]	5.4 ± 1.0 [89.2 ± 17.0%]	0.024
	*n* (%) with score <80%	26 (54.2%)	12 (25%)	0.003
Crisis resources management	Original score (full score* *= 2–4) [%]	3.2 ± 1.2 [86.5 ± 26.3%]	1.7 ± 1.3 [59.4 ± 38.5%]	<0.001
	*n* (%) with score <80%	15 (31.3%)	31 (64.6%)	0.001
Behavioral skills^b^	Original score (full score* *= 8–10) [%]	9.0 ± 1.3 [93.2 ± 9.0%]	6.7 ± 1.5 [75.9 ± 12.8%]	<0.001
	*n* (%) with score <80%	5 (10.4%)	28 (58.3%)	<0.001

Data are presented as mean ± SD or *n* (%).

^a^ *N *= 48 and 35 participants in residents and HCPs groups, respectively, were assessed in advanced ISSA.

Crisis resources management: call for additional help if needed, and or identify additional interventions as indicated on history and clinical response to resuscitation.

^b^ Behavioral skills: preparation (know the environment, use available information, anticipate and plan, clearly identify a team leader) and crisis resources management.

After neo-RST workshops, residents group had higher overall ISSA score than that of HCPs group (90.2 ± 5.9% vs. 86.3 ± 6.6%, respectively, *P *= 0.003) with no significant difference in the numbers of participants scored <80% (3 [6.3%] vs. 7 [14.6%], respectively). Among 17 nurses and midwives in HCPs group, 4 of them opted for advanced ISSA. Regarding the categorical scores, residents scored higher in preparation, CRM and behavioral skills when compared with the HCPs (*P *< 0.001, *P *< 0.001, *P *< 0.001, respectively) (Table 2). The residents scored higher in advanced (PPV, MR SOPA and intubation) but not basic (PPV and MR SOPA) ventilation (*P *= 0.024 and *P *= 0.140, respectively). More participants in HCPs group had basic ventilation score <80% as compared with residents group (10 [20.8%] vs. 3 [6.3%], *P *= 0.037, respectively]. The HCPs group scored higher in appropriate oxygen use than residents (89.2% vs. 81.3%, *P *= 0.024, respectively). There was no significant difference in categorical scores in initial steps, intubation, chest compression and medication (Table 2).

The characteristics of pediatric residents (*n* = 34) and non-pediatric residents (*n* = 14) were further compared. The pediatric residents had more working experience than non-pediatric residents (2.0 ± 0.5 years vs. 1.6 ± 0.5 years, *P *= 0.015, respectively). Twenty-six percent of pediatric residents had prior training in neo-RST, whereas none of non-pediatric residents had prior training (*P *= 0.043). The non-pediatric residents had lower neonatal resuscitation knowledge scores (67.8 ± 15.7% vs. 79.5 ± 11.3% of pediatric residents, *P *= 0.006). After training, pediatric residents and non-pediatric residents had similar overall ISSA scores (90.7 ± 5.8% vs. 89.2 ± 6.2%, *P *= 0.455, respectively) (Table 3).

**Table 3:** Demographic characteristics and neonatal resuscitation performance of 34 pediatric residents and 14 non-pediatric residents.

	Pediatric residents	Non-pediatric residents	*P-*value
Work experience (year)	2.0 ± 0.5	1.6 ± 0.5	0.015
Delivery room available	28 (82%)	13 (93%)	0.656
Female gender	28 (82%)	5 (62%)	0.246
Previous participation in neonatal resuscitation simulation training	9 (26%)	0 (0%)	0.043
Neonatal resuscitation knowledge prior to workshop (scores in %)	79.5 ± 11.3	67.8 ± 15.7	0.006
Overall ISSA score (%) in neonatal resuscitation workshop (first assessment)	90.7 ± 5.8	89.2 ± 6.2	0.455
*n* (%) with overall ISSA score <80%	2 (5.9%)	1 (7.1%)	1.000

Data are presented as mean ± SD or *n* (%).

## Discussion

To our knowledge, this is one of the first studies to evaluate the feasibility and effectiveness of training residents neonatal resuscitation skills by two-day neo-RST workshops in China, using the Canadian NRP® training format, curriculum, evaluation and exit system in the context of Chinese medical educational system. We found that through standardized simulation training, residents could have the same short-term educational achievements (competence in simulated neonatal resuscitation assessment) as the more experienced HCPs.

Providers need to possess cognitive, technical and behavioral skills to effectively perform neonatal resuscitation (18). The pre-training test showed cognitive skills of residents were limited including pediatric residents. This might indicate inadequate neonatal resuscitation training previously. The neo-RST workshop proceeded training sequentially from cognitive to psychomotor phase and from two-provider resuscitation to team resuscitation. We set objectives of cognitive, technical and behavioral skills from skill stations and integrated the goals into simulation and debriefing (Table 1). Compared with traditional education methods, simulation has been proven to better prepare providers with cognitive, technical, and behavioral skills in neonatal resuscitation (19).

The fundamental of our workshop is consistent with learn-see-practice-prove-do-maintain (LSPPDM) pedagogical framework (20, 21). The participants learned through self-study of NRP® textbook and didactic lecture (learn), and observed skills performed by instructors in various skill stations (see). These two steps are considered as cognitive phase. After that, trainees practiced maneuvers in skill stations (practice), then integrated and performed simulated resuscitation in ISSA to prove competence (prove). After the workshop, they were encouraged to perform neonatal resuscitation in clinical settings (do) and to maintain neonatal resuscitation skills during clinical practice (maintain). These four steps are considered as psychomotor phase. Repeated and dedicated practice are needed until the learner is competent for the resuscitation skills.

Positive pressure ventilation is one of the most important and essential skills in neonatal resuscitation (18, 22). The result of better performance in basic and advanced ventilation scores by residents (less participants with scores <80% and higher original score in advanced ventilation, Table 2) in our study is interesting. In China, experienced HCPs in NICU tended to perform endotracheal intubation early without completion of corrective ventilation steps or intubated a baby immediately after birth if the heart rate was low (personal observation). In addition to the differences in health care systems, educational and training backgrounds of HCPs between developed countries and China, it is difficult to change their cognitive skills of PPV and MR SOPA formed over years in a short period of 2-day workshops. Therefore, it is crucial for residents to learn standard resuscitation skills in the early stage of their career. Of note, we also found that residents were competent in ventilation skills after simulation training with scores of 91%.

The residents also scored significantly higher in CRM and behavioral skills than HCPs in our study. The residents tended to call for help more promptly after intubation or chest compression. In contrast, HCPs were more willing to handle the situation by themselves without additional assistance. The residents might be more adaptable to formal assessment which always included preparation in standardized resident training program. Nevertheless, this may also reflect that behavioral skills could be mastered quickly within a short period of training. However, it must also be acknowledged that the low total score of CRM in ISSA might have led to bias in the evaluation of HCPs.

In contrast, the higher scores in appropriate oxygen use of HCPs group may reflect that they were more experienced and cautious about the side effect of oxygen use. The use of room air in neonatal resuscitation and the awareness of adverse effects of oxygen overuse have been advocated since 2006 in China and the 5th edition of NRP® textbook (23, 24). Further HCPs tended to be more sensitive to adjust the fraction of inspired oxygen timely based on targeted oxygen saturation.

Competency-based training has been widely adopted in medical education in recent years, shifting towards an outcome-based rather than time-based approach (25–27). Six core competences that residents should possess have been identified by China Consortium of Elite Teaching Hospital for Residency Education in 2018, including professionalism, medical knowledge and skill, patient care, communication and collaboration, teaching and life-long learning (28). Different from didactic teaching, simulation-based training includes technical skills, teamwork and communication skills (19). NRP® key behavioral skills (15) were reinforced during team training. The trainees practiced to know the environment, use available information, anticipate and plan, clearly identify a team leader, communicate effectively, delegate workload optimally, allocate available resources wisely, allocate attention wisely, call for additional help if needed and maintain professional behavior during the team training. Neonatal resuscitation is a dynamic process. Residents should be able to make decisions timely in emergency situation or work as the team leader to direct nurses and other teammates to solve current problems. They practiced to organize, plan and coordinate in an orderly manner. This training model caters to the requirements of competency-based medical education and will result in the mastery of core competencies necessary for neonatal resuscitation of qualified residents. Further the participation of residents in the team resuscitation will not only prepare them to take on leadership roles in the future, but also helps residents to collaborate and communicate with team members in neonatal resuscitation. Almost no participant could perform perfectly with full overall ISSA score. The goal of simulation training is not to cultivate perfect trainees, but to let them know how to learn from mistakes.

In addition to pediatric residents, the workshop also enrolled residents from obstetrics and gynecology, anesthesia and emergency departments. We assumed that non-pediatric residents also had opportunities to perform neonatal resuscitation under limited resources settings. Previously, Parotto et al. reported the efficacy of the NRP® course on knowledge (71-item questionnaire) retained by pediatric, anesthesia and gynecology residents (29). Hereby, the neo-RST workshops were well received by non-pediatric residents, which showed significant achievements in educational outcomes. Simulation-based workshops should further be explored in other departments and centers.

It must be acknowledged that the skills acquired in neo-RST workshops do not equal to clinical competence (20). Completion of NRP® does not necessarily imply competence in the clinical setting. Each hospital is responsible for determining the level of competence and qualifications of neonatal resuscitation providers (30). We believe that the neo-RST workshops equipped residents with concepts and basic skills of neonatal resuscitation. Following the LSPPDM pedagogical framework (20, 21), the residents were encouraged to practice and maintain the resuscitation skills after workshops. The experience of neonatal resuscitation in delivery room among residents varied widely. Residents' self-confidence level in leadership, intubation and umbilical venous catheterization skill was proportional to the frequency they participated in the resuscitation in delivery room (31). Adherence to the NRP® algorithm is challenging, and errors occur during neonatal resuscitation frequently and repeatedly (32). As neonatal resuscitation skills may deteriorate after training (33–35), it is suggested that regular refresher training is required (35). Continuous practice and education in delivery room is important. In a prospective observational study, the implementation of educational program in delivery room had improved the leadership of residents (36). In HKU-SZH, senior neonatal physicians would take the NICU residents to attend deliveries of various risk levels. In low-risk deliveries, the resident could be the team lead under supervision. In complicated cases, the resident could be the team member responsible for preparation, medical recording and other auxiliary tasks. The residents learned resuscitation skills to ensure optimal patient care and were prepared to work under the supervision of senior physicians. They would continue to practice the skills in clinical work and receive further training. Further, it remains unknown if the short term learned skill among the residents persists into a long-term practice. Weekly multidisciplinary in-situ simulation training involving residents in our center was associated with decreased incidence of asphyxia, severe asphyxia, hypoxic-ischemic encephalopathy and meconium aspiration syndrome (37). Medical training, from undergraduate to resident and beyond with continuing medical education, will benefit the individual if each stage is closely linked who also fully understands needs, trends and changes (38).

### Challenges

The prevalence of neo-RST workshops among residents in China is low. In our study, only 19% of all residents (including 26% among pediatric residents) had previous neonatal resuscitation training. Uneven distribution of medical and educational resources among various regions in China still exits and should be considered in the education of residents on neonatal resuscitation. Developing pediatric residents with qualified skills remains challenging. Collaboration between training centers/organizations will help ensure the competency of pediatric residents and provide safe care to patients and families (39). Development of regional simulation centers and up-to-date faculty training may gradually benefit all residents. Nevertheless, the short-term educational outcomes are encouraging. Longitudinal data is needed to test the sustainability and flexibility of neo-RST workshops in China. Training centers need to follow and conduct continuous assessment of residents' performance in clinical settings as appropriate.

### Limitations

There are several limitations in our study. Firstly, our sample size is small, and the results need to be validated in other training centers. Secondly, we utilized ISSA with standardized format without much consideration of clinical reasoning and special circumstances, which may affect the performance of HCPs. The evaluation tool is a pass/fail assessment rather than a graded evaluation on the ability or competence of trainees. Further ISSA focuses more on technical skills than on team performance. Thirdly, we only evaluated short-term educational outcomes without the assessment of performance in real clinical situation. Fourthly, it was impossible to blind the ISSA scoring because two workshops were exclusively designed for residents. While the instructors were experienced HCPs who were certified through institutional neo-RST instructor workshops, a group of 6 congruent instructors participated in all 4 neo-RST workshops to minimize bias in data acquisition.

In summary, neo-RST for residents, a formal workshop that adopts NRP® training format, curriculum, evaluation and exit system in 2 days, is feasible with promising short-term educational outcomes. Simulation-based resuscitation training could be implemented in more training centers of the standardized medical residency programs. Despite the challenges, NRP® (8th Edition) training curriculum needs to be included and mandated for residency in Pediatrics and allied branches not only in the developed countries but globally in all birthing places to control the morbidity and mortality due to hypoxic ischemic neonatal injury.

## Data Availability

The raw data supporting the conclusions of this article will be made available by the authors, without undue reservation.
