# Supplementary material for: Neonatal resuscitation workshop for trainees in standardized medical residency training—a pilot practice in Shenzhen, China
Source: Front Pediatr. 2023 Sep 4;11:1237747. doi: 10.3389/fped.2023.1237747 (PMC10512178; doi:10.3389/fped.2023.1237747)
Supplement: Supplementary file 1 [file Datasheet1.pdf]

Individual Integrated Skills Station Assessment Form - Canadian Adaptation

Canadian Paediatric Society / Société canadienne de pédiatrie

BASIC ISSA

Learner:	Date:	Evaluator:	

SCORING: 0= Not done 1= Done incorrectly, incompletely or out of order 2= Done correctly in order

Brief:

- ✓ Learners must perform each of the **6 bolded and shaded** items correctly.
- ✓ **Bolded** (but not shaded) item is often missed clinically and instructors should emphasize its importance.
- ✓ Learners will be evaluated according to their role and scope of practice. For example, if the skill is not within the learner's scope, he/she will be evaluated in the 'assist' role.
- ✓ If the skill is not scored (i.e. consider intubation), the Instructor may provide additional feedback and instruction on these skills.

Lesson	Possible Points	Item	0	1	2	Comments
2	2	Asks 4 pre-birth questions *(Expected GA, Fluid clear, #Fetuses, Risk factors)*				
2	2	Discusses plan and assigns roles to team members				
2	2	**Checks equipment to provide warmth, suction, ventilation and targeted oxygenation**				
	2	Asks 3 assessment questions *(Term, Tone, Breathing or Crying)*				
3	2	Positions head, clears airway if necessary				
	2	Dries[1], stimulates and removes wet towels and repositions head				
	2	Assesses respirations +/- heart rate[2]				
4	2	**Indicates need for and initiates positive-pressure ventilation**[3] *(Apnea or gasping, heart rate<100 bpm)*				
	2	Checks for rising heart rate after 15 seconds of PPV **(Instructor note: Heart rate does not improve and chest is not moving)*				
	2	**Takes corrective action when heart rate not rising & chest not moving** *(Mask readjustment, **R**eposition; **S**uction mouth & nose, **O**pen mouth; **P**ressure increase; **A**lternate airway)*				
	No Score	If unable to ventilate through ET tube ('A' of MR.SOPA) indicates option to suction through ET tube or use ET to suction below the cords	**No Score**			
	No Score	Confirms presence of chest movement, breath sounds and exhaled CO_2 if intubated or LMA in situ	**No Score**			
	2	Initiates monitoring[4] *(pulse oximeter probe to right wrist or hand +/- ECG)*				
	2	Calls for help, if not already done				
	2	**Provides effective positive pressure ventilation** *(40-60 bpm)* **for 30 seconds**				
	2	Re-evaluates heart rate **(Instructor note: Heart rate must remain <60 bpm)*				
5	No Score	Consider intubation if not already done	**No Score**			
	2	Demonstrates correct technique for intubation or assisting with intubation				
	No Score	Confirms presence of chest movement, breath sounds/air entry and exhaled CO_2	**No Score**			
6	2	**Identifies need to start chest compressions** *(Heart rate <60 bpm despite 30 seconds of effective positive pressure ventilation)*				
	2	**Increases oxygen to 100% when initiating compressions**				
	2	**Demonstrates correct compression technique for 60 seconds** *(2-thumb method, compression depth 1/3 anterior-posterior diameter, complete recoil of chest)*				
	2	Demonstrates correct rate and coordination with ventilation *(Can ask student and assistant to switch positions)*				
	2	Administers blended oxygen to meet targeted saturations using pulse oximeter during resuscitation sequence				Do not score if completing Advanced ISSA
Closure	2	Continues/discontinues positive-pressure ventilation appropriately or weans oxygen correctly				Do not score if completing Advanced ISSA
		Learner's score subtotals				
End of **BASIC** ISSA<br><br>**ADVANCED** ISSA on back of page		Learner's total score (add subtotals)				
		Total possible score BASIC ISSA (42)<br>*If learner is completing the ADVANCED ISSA maximum score is 38 as last 2 items of BASIC ISSA are not scored above*				
		Performed all **6 bolded & shaded** items correctly? Yes ☐ No ☐	Re-evaluate ☐			
		Learner attained minimum passing score?<br>**MINIMUM PASSING SCORE is 35/42**	Y Pass<br>N Re-evaluate			

Individual Integrated Skills Station Assessment Form - Canadian Adaptation

Canadian Paediatric Society / Société canadienne de pédiatrie

ADVANCED ISSA

Lesson	Possible Points	Item	0	1	2	Comments
		**BASIC ISSA total** (from page 1)				
7	2	Identifies need for epinephrine<br>*Heart rate <60 bpm despite PPV and compressions for 60 seconds*				
	2	Identifies correct dose and route for epinephrine *(0.1mL/kg IV and 1.0mL/kg ET to maximum of 3mL/dose)*				
	No score	Administers ET dose while umbilical catheter being prepared	No score			
		Prepares or assists with preparation of umbilical catheter for insertion				
		Inserts or assists with insertion of umbilical venous catheter				
		Administers epinephrine via umbilical venous catheter				
		Indicates option to insert intraosseous needle if unable to place an umbilical venous catheter				
	2 (optional)	Identifies need for volume administration and administers correct solution, volume and rate of infusion				
8-11	2 (optional)	Identifies additional interventions indicated based on history and clinical response to resuscitation<br>*(For example, care of an extremely preterm infant, infant with a pneumothorax, diaphragmatic hernia, etc.)*				
	2	Administers blended oxygen to meet targeted saturations using pulse oximeter during resuscitation sequence				
Closure	2	Continues/discontinues positive-pressure ventilation appropriately or weans oxygen correctly				
		Learner's score subtotals (page 2)				
		Learner's total score (add subtotals)				
		Total possible score (46 without optional skills; 48 with 1 optional skill or 50 with 2 optional skills)				
		Performed all **6 bolded & shaded** items correctly? Yes ☐ No ☐	Re-evaluate ☐			
		Learner attained minimum passing score?<br>**MINIMUM PASSING SCORE IS 39/46; 40/48 or 42/50**	Y Pass<br>N Re-evaluate			

**Learner Self Reflection**<br>*What went well in this resuscitation?*	**Learner Self Reflection**<br>*What would you do differently?*	**Instructor Feedback**
Instructor Signature:	☐ Pass ☐ Re-evaluate (x1 and pass) ☐ Unsuccessful – advised to retry after review<br>If re-evaluation by a different instructor, please indicate name, date and outcome: ______________________	

1) Drying the skin does not apply to babies <32 weeks; they should be placed wet into a food-grade polyethylene bag below the neck.
2) Heart rate may be assessed by auscultation or ECG; respirations may be assessed by chest movement or by auscultation.
3) For term infants, begin PPV with 21% oxygen; for infants <35 weeks GA, follow local protocols.
4) PPV and assessment of HR are the priority and should not be unduly delayed by the application of a pulse oximeter probe.
