# Supplementary material for: Neonatal resuscitation workshop for trainees in standardized medical residency training—a pilot practice in Shenzhen, China
Source: Front Pediatr. 2023 Sep 4;11:1237747. doi: 10.3389/fped.2023.1237747 (PMC10512178; doi:10.3389/fped.2023.1237747)
Supplement: Supplementary file 2 [file Datasheet2.pdf]

Basic ISSA 新生儿复苏考核表（基础版）

姓名：_______　　　　　　得分：_______

	0	1	2	Comments
4个问题：胎龄，羊水，单/双胎，高危因素				
与团队成员讨论计划、分配任务				
检查设备：保暖、吸引、通气、目标氧合				
问3个问题：足月，肌张力，呼吸或哭声				
摆好体位（head position），必要时吸引				
擦干，刺激，移调湿毛巾，重新摆好头位				
评估呼吸±心率				
识别PPV指征并开始实施（呼吸暂停或喘息样呼吸，心率<100次/分）				
15秒PPV后评估心率有无上升，胸廓起伏				
矫正通气MR.SO.P.A(如心率无上升，胸廓无起伏)				
如果ETT后无法通气，有指征经ETT吸引	No Score			
插管后确认有胸廓起伏，双肺呼吸音，呼出CO2	No Score			
开始监护（SpO2±EEG）				
呼叫帮助				
有效PPV(40-60次/分)30s				
再次评估心率				
插管时操作正确及助手配合				
胸外按压指征正确识别（有效正压通气30s后心率仍<60次/分）				
胸外按压时FiO2 100%				
正确胸外按压60s（后评估心率）				
胸外按压频率正确，与通气配合				
使用空氧混合仪，根据目标氧饱和度目标调节氧浓度				
根据病情继续/停止PPV，正确下调氧浓度				
总分				

灰色部分为6项核心技能

Advanced ISSA 新生儿复苏考核表（进阶版）

<table>
<tr><th></th><th>0</th><th>1</th><th>2</th><th>Comments</th></tr>
<tr><td>肾上腺素指征正确（有效 PPV，胸外按压 60s 后心率仍<60bpm）</td><td></td><td></td><td></td><td></td></tr>
<tr><td>肾上腺素剂量正确（0.1ml/kg iv，1ml/kg 经 ETT 最大剂量 3ml）</td><td></td><td></td><td></td><td></td></tr>
<tr><td>UVC 正在准备时，ETT 使用肾上腺素</td><td colspan="3" rowspan="5">No Score</td><td></td></tr>
<tr><td>准备 UVC</td><td></td></tr>
<tr><td>经 UVC 使用肾上腺素</td><td></td></tr>
<tr><td>考虑骨髓穿刺给药（无法放置 UVC 时）</td><td></td></tr>
<tr><td>识别扩容指征，剂量，时间</td><td></td></tr>
<tr><td>根据病史、临床表现，识别可能需要的额外治疗（早产儿、气胸、膈疝）</td><td></td><td></td><td></td><td></td></tr>
<tr><td></td><td></td><td></td><td></td><td></td></tr>
</table>

说明：

1. 32 周以下早产儿不需要擦干，在颈部以下放置塑胶袋。
2. 评估心率：听诊或 EEG；评估呼吸：胸廓起伏或听诊。
3. 用氧：足月儿 21%；小于 35 周早产儿：21-30%。
4. PPV 和评估心率是最首要的/priority，不应延迟到放置 SpO_2 之后进行。

考核人：_________
